# (+)-9-Trifluoroethoxy-α-Dihydrotetrabenazine as a Highly Potent Vesicular Monoamine Transporter 2 Inhibitor for Tardive Dyskinesia

**DOI:** 10.3389/fphar.2021.770377

**Published:** 2021-12-07

**Authors:** Wenyan Wang, Guangying Du, Shilan Lin, Jing Liu, Huijie Yang, Dawei Yu, Liang Ye, Fangxia Zou, Hongbo Wang, Rui Zhang, Jingwei Tian

**Affiliations:** ^1^ School of Pharmacy, Key Laboratory of Molecular Pharmacology and Drug Evaluation (Yantai University), Ministry of Education, Collaborative Innovation Center of Advanced Drug Delivery System and Biotech Drugs in Universities of Shandong, Yantai University, Yantai, China; ^2^ New Drug Discovery and Research Department, R&D Center, Luye Pharma Group Ltd., Yantai, China; ^3^ Department of Clinical Medicine, Binzhou Medical College, Yantai, China

**Keywords:** vesicular monoamine transporter 2, dihydrotetrabenazine, DA uptake, tardive dyskinesia, metabolite identification, metabolic enzyme phenotype

## Abstract

Valbenazine and deutetrabenazine are the only two therapeutic drugs approved for tardive dyskinesia based on blocking the action of vesicular monoamine transporter 2 (VMAT2). But there exist demethylated inactive metabolism at the nine position for both them resulting in low availability, and CYP2D6 plays a major role in this metabolism resulting in the genetic polymorphism issue. 9-trifluoroethoxy-dihydrotetrabenazine (13e) was identified as a promising lead compound for treating tardive dyskinesia. In this study, we separated 13e via chiral chromatography and acquired *R,R,R*-13e [(+)-13e] and *S,S,S*-13e [(−)-13e], and we investigated their VMAT2-inhibitory activity and examined the related pharmacodynamics and pharmacokinetics properties using *in vitro* and *in vivo* models (+)-13e displayed high affinity for VMAT2 (K_i_ = 1.48 nM) and strongly inhibited [^3^H]DA uptake (IC_50_ = 6.11 nM) in striatal synaptosomes. Conversely, its enantiomer was inactive. *In vivo*, (+)-13e decreased locomotion in rats in a dose-dependent manner. The treatment had faster, stronger, and longer-lasting effects than valbenazine at an equivalent dose. Mono-oxidation was the main metabolic pathway in the liver microsomes and in dog plasma after oral administration, and glucuronide conjugation of mono-oxidized and/or demethylated products and direct glucuronide conjugation were also major metabolic pathways in dog plasma. O-detrifluoroethylation of (+)-13e did not occur. Furthermore, CYP3A4 was identified as the primary isoenzyme responsible for mono-oxidation and demethylation metabolism, and CYP2C8 was a secondary isoenzyme (+)-13e displayed high permeability across the Caco-2 cell monolayer, and it was not a P-glycoprotein substrate as demonstrated by its high oral absolute bioavailability (75.9%) in dogs. Thus, our study findings highlighted the potential efficacy and safety of (+)-13e in the treatment of tardive dyskinesia. These results should promote its clinical development.

## Introduction

Tardive dyskinesia (TD) is a movement disorder occurring after long-term treatment with dopamine antagonists such as typical or atypical antipsychotics and certain antidepressants (Albayrak and Ekinci, 2012; Waln and Jankovic, 2013). The prevalence of TD remains substantial in patients treated with atypical (20%) and conventional antipsychotic agents (30%) (Carbon et al., 2017). Although the pathophysiology of TD remains to be fully elucidated, it has been commonly accepted since the 1950s that excessive dopamine accumulation plays a role in TD (Sarva and Henchcliffe, 2018). To date, the relationship between altered dopaminergic neurotransmission and TD remains a principal focus of research (Black et al., 2021; Farber et al., 2021).

Vesicular monoamine transporter 2 (VMAT2), expressed in the neuronal cells of the central nervous system and sympathetic adrenal chromaffin cells, is responsible for the uptake of cytosolic monoamines, including serotonin, norepinephrine, histamine, and dopamine, into synaptic vesicles in monoaminergic neurons (Liu and Edwards, 1997; Wimalasena, 2011; Lohr et al., 2017). VMAT2 inhibitors block the action of VMAT2 and inhibit its activity, thereby reducing the uptake and storage of monoamines from the cytoplasm to presynaptic vesicles and resulting in reduced concentrations of monoamines, which counteracts the increased activity of the dopamine system (Mulvihill, 2019).

**FIGURE 1:**
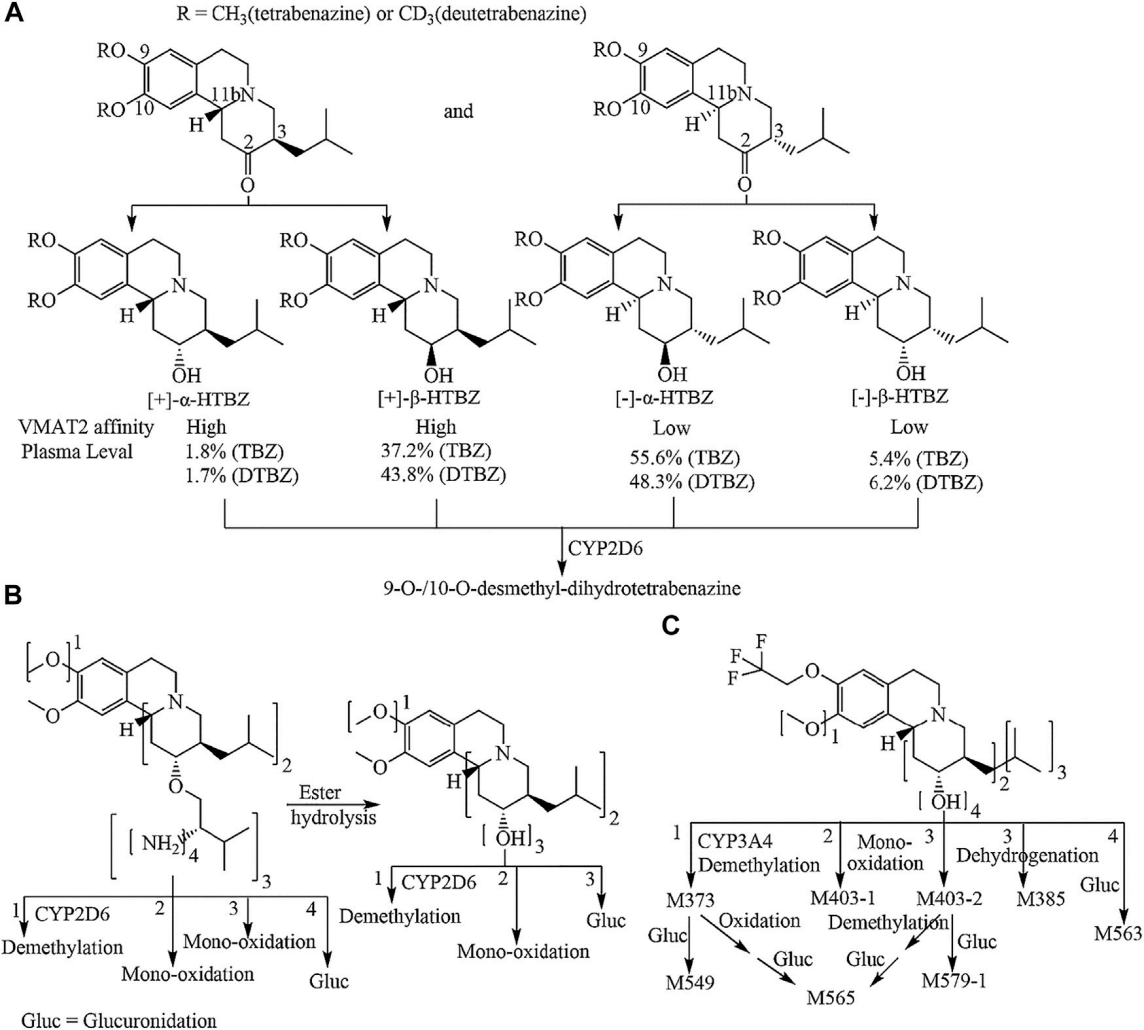
Metabolic pathways of tetrabenazine and deutetrabenazine **(A)** valbenazine **(B)** and (+)-9-trifluoroethoxy-α-dihydrotetrabenazine [(+)-13e] **(C)**. Note: the numbers 1, 2, 3 and 4 in B and C are marked outside the square brackets of the chemical group to indicate the metabolic pathway of the corresponding chemical sites.

Valbenazine (VBZ) and deutetrabenazine (DTBZ) were approved by the FDA for the treatment of TD in 2017 (Kim, 2017; Cummings et al., 2018) Both drugs, which are based on tetrabenazine (TBZ) pharmacology, block the action of VMAT2 (Dorfman and Jimenez-Shahed, 2021). DTBZ is a form of TBZ deuterated at the methoxy groups at the 9 and 10 positions. Although deuteration prolongs the half-life of DTBZ, it is inevitable that produces four dihydrotetrabenazine (HTBZ) isomer metabolites (Figure 1A), and (−)-α-HTBZ and (−)-β-HTBZ account for half proportion which may contribute to unwanted side effects (Skor et al., 2017; Stahl, 2018). VBZ is an ester prodrug of [+]-α-HTBZ which is the most potential and selective VMAT2 inhibitor among TBZ metabolite isomers identified to date, but because of the slow ester hydrolysis and its extensive metabolism (Figure 1B), the percent molar ratio of (+)-α-HTBZ (hereafter HTBZ) in healthy male subjects is 14.8–18.7% after treatment at a dose of 50–100 mg (Müller, 2015; Luo et al., 2017). Moreover, CYP2D6 plays a major role in the formation of demethylated inactive metabolites for all four dihydrotetrabenazine isomers (Schneider et al., 2020).

In our previous study (Yang et al., 2021), the promising lead compound 9-(2,2,2-trifluoroethoxy)-dihydrotetrabenazine (13e), with high affinity for VMAT2 (IC_50_ = 5.13 nM) and strong inhibitory effects on [^3^H]DA uptake in striatal synaptosomes, was identified. In view of the existence of potential unwanted side effects from other isomers, we performed the chiral resolution of 13e and acquired the (*R,R,R*) configuration isomer of (+)-9-(2,2,2-trifluoroethoxy)-α-dihydrotetrabenazine [(+)-13e]. Furthermore, the pharmacodynamic (PD) and pharmacokinetic (PK) properties of (+)-13e were investigated *in vivo* and *in vitro*.

## Materials and Methods

### Materials

#### Chemicals and Reagents

13e (3-isobutyl-10-methoxy-9-(2,2,2-trifluoroethoxy)-1,3,4,6,7,11b-hexahydro-2H-pyrido [2,1-a]isoquinolin-2-ol) (purity >98%) was offered by the Medicinal Chemistry Research Department, R and D Center (Luye Pharma Group Ltd., Yantai, China). Valbenazine ditosylate (VBZ) and its metabolite HTBZ (purity >99%) was purchased from Jiangsu Weikeer Pharmaceutical Technology Co., Ltd. HPLC-grade acetonitrile and methanol was purchased from Merck (Darmstadl, Germany). Human, rat and dog liver microsomes and recombinantly expressed human cytochrome P450 (rhCYP) isozymes were purchased from Corning Co., Ltd (Shanghai, China). Substrates and their specific metabolites and the specific inhibitors and NADPH were purchased from Sigma-Aldrich (Shanghai, China). All chemicals and reagents were of analytical grade and purchased from Sinopharm Chemical Reagent (Shanghai, China).

#### Animals

The adult male Sprague Dawley (SD) rats (weighing 200 ± 20 g) and healthy male beagle dogs (weight 8–9 kg) were obtained from the Experimental Animal Center of Luye Pharma Group Ltd., Yantai, China (Yantai, Shandong, China) and were housed were housed in the controlled environment including temperature (21 ± 2°C), relative humidity (50 ± 10%) and 12-h light/dark cycle. All animals had free access to feed and water in strict accordance with the National Institute of Health Guide for the Care and Use of Laboratory Animals (NIH Publications No. 8023). And the experiment protocols were approved by Institutional Animal Care and Use Committee.

### Methods

#### Resolution of 13e Isomers by Chiral Chromatography

According to the literature (Yao et al., 2011), 13e was chirally separated using (1S)-(t)-10-camphorsulfonic acid [(+)-CSA] as a resolving agent to acquire (+)-13e. When the equivalent of (+)-CSA was used, a yield of approximately 33% yield obtained from after one-time recrystallization of the (+)-CSA•(+)-13e salt from acetonitrile, and using (−)-CSA in place of (+)-CSA as a resolving agent, (−)-13e was obtained using the same methodology. The purity of each isomer was determined using a Chiralpak AD-H HPLC column (0.46 cm I.D. × 15 cm L, Daicel, Japan) eluted by hexane:ethanol (60:40, v/v) at a flow rate of 1 ml/min. Then, their tosylates were prepared and cultured as single crystals in methanol. The crystalline samples were subjected to X-ray crystal structure analysis using a Rigaku Oxford Diffraction XtaLAB Synergy four-circle diffractometer equipped with a HyPix-6000HE area detector.

#### PD Studies

##### VMAT2 Binding Assay

Using the previously reported method (Teng et al., 1998), the inhibition of the binding of [^3^H]HTBZ ((±)-α-[O-methyl-^3^H]dihydrotertabenazine; specific activity, 20.0 Ci/mmol) was accomplished by Eurofins Panlabs (Taipei, Taiwan). In brief, the whole rat brain (excluding the cerebellum) or striatal were homogenized in 20 ml of ice-cold sucrose solution (0.32 M). The homogenate was centrifuged at 1,000 g at 4°C for 12 min, then the supernatant was centrifuged again at 22,000 g at 4°C for 10 min. And the pellets were resuspended and incubated in 18 ml of ice-cold MilliQ water for 5 min, then added 2 ml of HEPES (25 mM) and 2 ml of potassium tartrate (100 mM) solution. The samples were centrifuged at 20,000 g at 4°C for 20 min, then 20 ml of MgSO_4_ solution (1 mM) was added to the supernatant. The mixed solution was centrifuged at 100,000 g for 45 min at 4°C and the pellets were resuspended in ice-cold assay buffer (25 mM HEPES, 100 mM potassium tartrate, 5 mM MgSO_4_, 0.1 mM EDTA and 0.05 mM EGTA, pH 7.5) to acquire the vesicle suspension.

A 96-well plate was used for the assay in duplicate. A 50 μl of vesicle suspension (640 μg protein/mL) containing 10 nM [^3^H]HTBZ, 50 μl analyte (1 nM–1 mM) and 50 μl assay buffer were added to each well. Samples were preincubated for 30 min at room temperature. Nonspecific binding was determined in the presence of 10 μM Ro4-1,284. In addition, HTBZ and TBZ was used as positive control. All reactions were terminated using vacuum filtration over a GF/B glass fiber filter pretreated with 0.5% BSA. The filters were then washed five times with 350 μl of ice-cold washing buffer (25 mM HEPES, 100 mM potassium tartrate, 5 mM MgSO_4_ and 10 mM NaCl, pH 7.5), followed by the addition of 40 μl of Microscint 20 (Perkin Elmer) to each filter. Radioactivity retained on the filters was determined by liquid scintillation counter. Specific [^3^H]HTBZ binding was determined by subtracting nonspecific binding from total binding. IC_50_ values were determined by a nonlinear least-squares regression analysis and K_i_ was calculated.

##### [^3^H]DA Uptake Assay

[^3^H]DA (dihydroxyphenylethyethylamine, 3,4-[ring-2,5,6-^3^H]; specific activity, 28.0 Ci/mmol) was purchased from Perkin Elmer. One microliter of diluted compounds (from 0.2 mM to 0.122 μM, fourfold serial dilution with DMSO) were transferred to the assay plates with final concentrations ranging from 1 μM to 0.61 nM, total eight concentration points in duplicate. HTBZ and TBZ were used as positive control. 1 μl of DMSO was then added as for total binding and 1 μl of 2 mM TBZ for nonspecific binding. Then, 100 μl of diluted vesicular suspension containing 15 μl of vesicular stock solution was added to 96-well plates, followed by incubation at 37°C for 15 min 100 μl of [^3^H]DA (0.2 mM, dilution with assay buffer) or vehicle control (0.1% DMSO) was added, followed by incubation at 37°C for 10 min. The mixture was then vacuum-filtered by Whatman GF/B filters using a Brandel harvester, followed by washing four times with cold buffer (25 mM HEPES, 100 mM potassium tartrate, 50 μM EGTA, 100 μM EDTA, pH 7.4). After drying, the filter plate was sealed with unifilter-96 back sealing tape and added 50 μl of microscint-20 (Perkin Elmer), then sealed the top of the filter plate with TopSeal-A. The radioactivity was measured by a TopCount scintillation counter. GraphPad Prism 5.0 was used to analyze data.

##### Dose-dependent Effects on Locomotor Activity in Rats

In this study, we utilized a rat model of monoamine depletion as a surrogate marker, and the decreased rat autonomous activity to test whether (+)-13e inhibits VMAT2 *in vivo* after a single oral gavage at different doses using VBZ as a control. SD rats were randomly assigned into nine treatment groups (n = 12 per group, 6/sex), including a control group [normal saline (NS)], VBZ group (5.2 μmol/kg), and seven (+)-13e dose groups (0.16, 0.32, 0.64, 1.3, 2.6, 5.2, and 10.4 μmol/kg). Before administration, animals were transported to the test room and allowed to acclimate for at least 1 h. Immediately after drug administration, rats were placed into activity chambers to assess their basal locomotor activity using the TopScan monitoring system. The total travel distance was recorded from 0.5 to 1.5 h after administration in intervals of 10 min. The reduction rate (RR) of locomotion was calculated as follows: RR = [(total distance traveled in the NS group − total distance traveled in the (+)-13e group)/(total distance traveled in the NS group)] × 100%.

##### Time-dependent Effects on Locomotor Activity in Rats

Based on the results of the aforementioned dose–effect relationship study, the time–effect relationship of (+)-13e was investigated at doses of 0.32 and 1.3 μmol/kg. In brief, in each period, the rats were randomly divided into four groups (12 rats per group, 6/sex), including the control group (NS), VBZ group, and two (+)-13e groups. Rats were placed into the detection box after a single intragastric administration at different time points, and two rats from each group were used in each round of the experiment. The TopScan monitoring system was used to record and analyze the locomotor activity of the rats in different periods (0–1, 1–2, 3–4, 5–6, 6–7, and 7–8 h) after administration, and the RR was calculated as previously described.

#### PK Studies

##### Bidirectional Permeability in Caco-2 Cell Monolayers

Caco-2 cells (ATCC, United States) were seeded on a 96-multiwell insert system (Corning, United States) at a density of 1.0 × 10^5^ cells/cm^2^. After about 24 days of culture, fluorescein permeability was measured to evaluate the integrity of Caco-2 cell monolayers, and the percent of lucifer yellow in basolateral well which should be less than 1%. Before initiating the transport studies, the cell monolayers were washed with warm HBSS. Then, HBSS containing (+)-13e (2, 10 and 100 μM) or digoxin (10 μM) was loaded into either the apical (AP) or basolateral (BL) chambers with or without verapamil (30 μM, P-gp inhibitor) for 2 h incubation at 37°C. Aliquots from the receiver chamber were collected at 2 h after incubation and stored at −20°C until analysis via LC-MS/MS. All experiments were conducted in triplicate. Permeability coefficient (P_app_) and the efflux ratio (ER = *P*_app_ (BL→AP)/*P*_app_ (AP→BL)) were calculated.

##### Metabolite Identification in Liver Microsomes of Different Species

Human, rat and dog liver microsomes were carefully thawed on ice prior to the experiment and diluted to 1 mg/ml in100 mM sodium phosphate buffer (pH 7.4) containing 10 mM magnesium chloride. Two microliters of the (+)-13e stock solution (1 mΜ in MeOH) were added to the tube, and after volatilizing to dryness, 180 µL of microsome proteins (1 mg/ml) were added. After 3 min of preincubation at 37°C, the reactions were initiated by adding 20 µL of NADPH (20 mM) and then incubated at 37°C in a shaking water bath for 60 min. All samples were prepared in duplicates. The reactions were terminated by adding 600 µl of ice-cold acetonitrile and stored at −20°C until analysis. Control samples without NADPH and 0-time point samples were included. As a positive control, testosterone (10 µM) which was metabolized to produce the hydroxylation metabolite was used as a substrate to confirm the activity of the incubation system. Next, the samples were centrifuged at 13,000 rpm for 10 min, the supernatant was separated and evaporated to dryness under a nitrogen stream. The residue was resuspended in 100 μl of acetonitrile, after centrifugation, the supernatants were analyzed by UPLC–UV-HRMS.

##### Identification of P450 Isozymes Responsible for Metabolism

According to the result of metabolite identification, three metabolites were selected as the targets for metabolic phenotyping. Seven CYP isozymes (CYP1A2, CYP2B6, CYP2C8, CYP2C9, CYP2C19, CYP2D6, and CYP3A4) were investigated using two kinds of methods: chemical inhibitors in human liver microsomes (HLMs) method (Experiment 1) and rhCYP isozymes method (Experiment 2).

Experiment 1: HLMs were thawed on ice and diluted in100 mM sodium phosphate buffer (pH 7.4) containing 3 mM magnesium chloride. Two microliters of the (+)-13e stock solution (0.1 mΜ) and 2 µl of specific inhibitors were added to the tube, and after volatilizing to dryness, 180 µl of microsome proteins (0.5 mg/ml) were added. After 10 min of preincubation at 37°C, the reactions were initiated by adding 20 µL of NADPH (10 mM) and then incubated at 37 °C in a shaking water bath for 30 min. The samples were prepared in triplicates. The reactions were terminated by adding 600 µL of ice-cold acetonitrile and stored at −20°C until analysis. Control samples without NADPH and 0-time point samples were included. As positive controls, the probe substrates of each P450 isozyme under the same conditions were tested, and specific inhibitors was added. Next, the samples were centrifuged at 13,000 rpm for 10 min, the supernatants were determined by LC–MS/MS. The degree of inhibition on the metabolism of (+)-13e is calculated by comparing the production of metabolites in the presence and absence of the inhibitor.

Experiment 2: Each rhCYP isozyme was thawed and diluted as above method. As positive controls, the probe substrates of each P450 isozyme were tested under the same conditions. The relative contribution rate of each isozyme to the metabolism of (+)-13e was evaluated in HLMs using the following formula (Davies and Morris, 1993; Hosea et al., 2009): Contribution_x_% = V_x_ × Abundance_x_ (pmol/mg protein)/Σ(V_i_ × Abundance_i_). V_x_ (µL/min/pmol) is the production rate of metabolites for each rhCYP isozyme.

##### PK in Dogs and Metabolite Identificationin Dog Plasma

Male beagle dogs were randomly divided into four groups. Two groups (four/group) received a single oral dose of (+)-13e or VBZ, respectively, at 3 μmol/kg and the other two groups received an intravenous injection of (+)-13e or VBZ, respectively, at 0.6 μmol/kg. The dogs were fasted for 12 h before treatment and 4 h after treatment, but they were granted free access to water. Blood was collected from the vein before administration and 0.083 (only for injection groups), 0.25, 0.5, 1, 2, 3, 4, 6, 8, 12 and 24 h, and placed it in heparinized tubes, and centrifuged it at 8,000 rpm for 10 min at 4°C to separate the plasma, which we stored at −20 °C until analysis. The plasma samples were pretreated by protein precipitation with acetonitrile and quantitated by the validated LC-MS/MS (Shimadzu Exion-Triple Quad 4500) method. The PK parameters of (+)-13e, VBZ and its metabolite HTBZ were calculated by non-compartmental analysis with WinNonlin software, (Version 6.3, Pharsight Corporation, United States).

Next, the dog plasma samples in the oral (+)-13e group were mixed according to their area under concentration-curve (AUC) contribution at each time point (Hop et al., 1998). Then, four volumes of acetonitrile (containing 0.1% formic acid) were added into the pooled plasma samples to precipitate the proteins. After vortex-mixing and centrifugation, the supernatants were dried under a nitrogen stream. Then, the residues were resuspended in 100 μl of acetonitrile/water (1:1, v/v) with 0.1% formic acid. After centrifugation, the supernatants were analyzed by UPLC–UV-HRMS for metabolite identification.

#### Analysis Methods

##### LC-MS/MS Analysis

The system consisted of an Exion UHPLC system (Shimadzu, Japan) and a Triple Quad 4500 mass spectrometer (AB Sciex, United States) with an electrospray ionization source. Multiple reaction monitoring in the positive ionization mode was applied to measure analytes. Chromatographic separation was performed on a Acquity BEH C18 column (50 × 2.1 mm i. d., 1.7 μm, Waters, United States). The mobile phase was composed of 0.1% FA in water (A) and 0.1% FA in acetonitrile (B), and the flow rate was 0.4 ml/min. The elution conditions were the following linear gradient: 0–0.4 min: 90% A, 0.4–0.6 min: 60% A, 0.6–1.2 min: 10% A, 1.2–2.2 min: 10% A, 2.2–2.3 min: 90% A, 2.3–3.0 min: 90% A. The optimized parameters were as follows: the spray voltage 5.5 kV, source temperature 550 °C, pressure of source gas 1 and gas 2: 55psi, pressure of curtain gas: 30psi, and the DP, EP and CE voltage was 30,6 and 40 eV, respectively. The transitions (precursor ion to product ion) monitored were *m/z* 388.2→344.2 for (+)-13e, *m/z* 419.3→302.1 for VBZ, *m/z* 320.4→274.2 for HTBZ, 13e, *m/z* 366.1→319.9 for internal standard.

The method was validated according to the relevant bio-analysis guidelines. There were no observed endogenous interference, carryover, or matrix effects for all analytes. The linearity of the method was 2–1,000 nM for (+)-13e, VBZ and HTBZ, and the intra-batch and inter-batch precision and accuracy met the acceptance criteria (CV% < 13.6%, RE% < 11.2%). The extraction recovery for all analytes ranged 66.9–78.9% (+)-13e was stable after incubation at room temperature for 4 h and in an autosampler (at 10°C) for 24 h. The dilution integrity of a 5-fold dilution was stable (RE% within ±6.4%, CV% ≤7.8%).

##### UPLC-Q Exactive™ Orbitrap HRMS Analysis

The analysis was performed on Acquity UPLC system coupled with a diode array detector (Waters Corporation, United States) and Q Exactive™ Orbitrap HRMS (Thermo Electron Corporation, United States). Samples were separated on an Agilent XDB C18 column (150 × 2.1 mm i. d. 3.5 μm, United States) at 40°C. The mobile phase was composed water containing 0.1% formic acid (A) and acetonitrile containing 0.1% formic acid (B). The flow rate was 0.40 ml/min. The elution conditions were the following linear gradient for metabolite identify in HLMs: 0–1 min: 95% A, 1–2 min: 80% A, 2–5 min: 80% A, 5–8 min: 70% A, 8–12 min: 30% A, 12–15 min: 5% A, 15–16 min: 95% A, and 16–18 min: 95% A. The elution conditions were the following for metabolite identify in dog plasma: 0–1 min: 95% A, 1–30 min: 70% A, 30–32 min: 30% A, 32–33 min: 5% A, 33–35 min: 95% A, and 35–40 min: 95% A.

HRMS were operated at positive ion mode using a heating electrospray ionization source (HESI). The optimized operating parameters were set as follows: sheath gas (nitrogen) flow rate of 35 psi, aux gas (nitrogen) flow rate of 10 psi, HESI and capillary temperature of 350°C, and spray voltage of 3.5 kV. The metabolites were detected by full MS/dd-MS^2^ analysis from *m/z* 100 to *m/z* 1,000. The resolution of the first-class scan and second-class scan was 70 000 and 17 500, respectively. The collision energy for collision induced dissociation (NCE) was set to 20, 40 and 60 eV. The loop count was set to five and the dynamic exclusion duration to 10 s. The ultraviolet (UV) detection wavelength was 286 nm. Thermo Compound Discover software was used to identify expected and unknown metabolites, and we finally proposed (+)-13e metabolic pathways based on the deduced metabolites and relevant drug biotransformation knowledge.

#### Statistical Analysis

The test data represented in figures are presented as the mean ± SEM. Analysis was performed using SPSS Statistics 20.0 software. One-way analysis of variance was used to compare the differences between the groups at each time point. All tests were two-sided, and *p* < 0.05 indicated statistical significance.

## Results

### Chemical Resolution of 13e and Configuration Analysis

**FIGURE 2:**
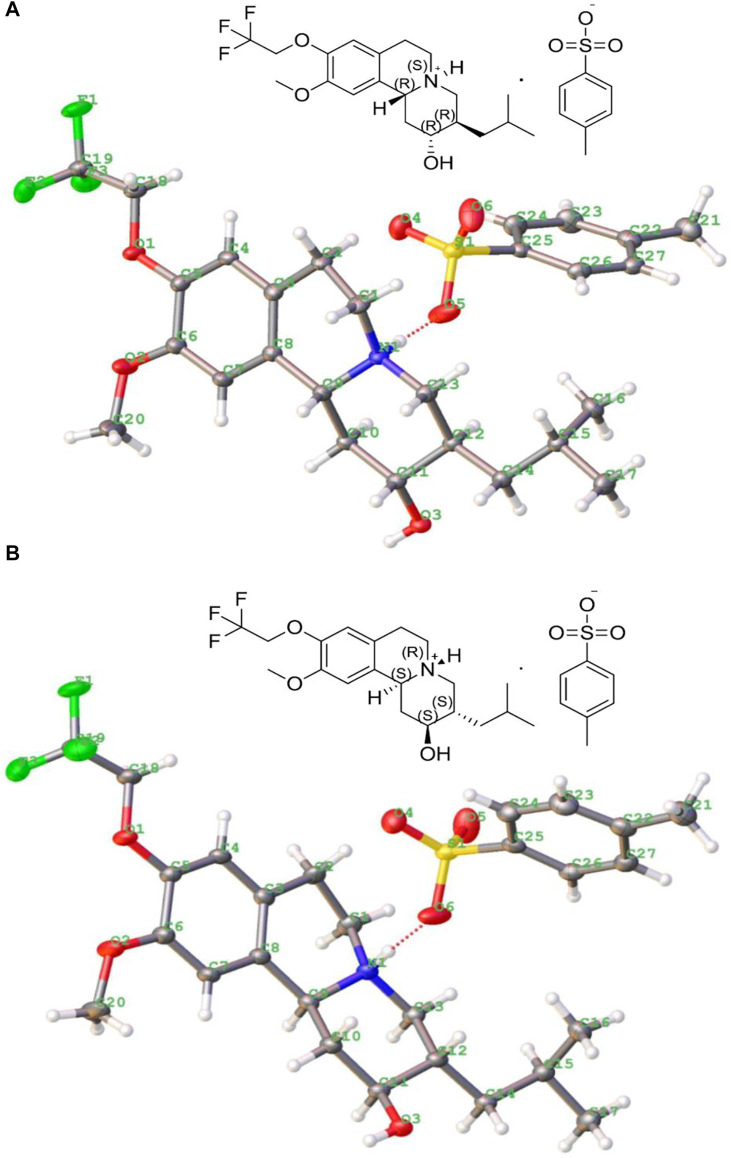
Crystal structures of *p*-toluenesulfonate of (+)-9-trifluoroethoxy-α-dihydrotetrabenazine [(2*R*,3*R*,11b*R*) isomer, (+)-13e] **(A)** and *p*-toluenesulfonate of (2*S*,3*S*,11b*S*) isomer [(−)-13e] **(B)**.

There are four isomer components of 13e with MS m/z (ESI) of 388.2087 [M + H]^+^, but two enantiomers accounted for the main relative abundance of 48.6 and 48.8%, respectively, and with purity >98.0%. The single crystals of these two main isomers’ *p*-toluenesulfonate were respectively grown from methanol solution via slow evaporation at room temperature (first raised the temperature to 40°C until the solid is completely dissolved, then the temperature of solution was reduced to 25°C with 0.3°C/min, stand a while). Both crystals formed colorless blocks, and the crystallographic data and refinement details are listed in Table 1, and their molecular ellipsoid diagrams are presented in Figure 2, which illustrated that one is the *p*-toluenesulfonate of (2*R*,3*R*,11b*R*) isomer, with the free base of (+)-9-(2,2,2-trifluoroethoxy)-α-dihydrotetrabenazine [(+)-13e] (DOI: 10.5517/ccdc.csd.cc28v81g, CSD Communication (CCDC deposition number 2110605). CCDC, Cambridge, England) and the other is the *p*-toluenesulfonate of (2*S*,3*S*,11b*S*) isomer [(−)-13e] (DOI: 10.5517/ccdc.csd.cc28v82h, CCDC deposition number 2110606). There were three rings in the isomer: one aromatic rings and two six-membered heterocyclic rings, and they did not share a common plane.

**TABLE 1:** Summary of X-ray Crystallographic Data for (+)-9-trifluoroethoxy-α-dihydrotetrabenazine [(+)-13e] and its enantiomer (−)-13e.

Compound	(+)-13e	(−)-13e
Crystal Size	0.20 × 0.10 × 0.10 mm^3^	0.20 × 0.20 × 0.10 mm^3^
Radiation Type	Cu Kα (*λ* = 1.54184Å)	Cu Kα (*λ* = 1.54184Å)
Crystal system	orthorhombic	orthorhombic
Space Group	P2_1_2_1_2	P2_1_2_1_2
Cell Size	a = 27.14408 (13) Å	a = 27.1619 (4) Å
	b = 16.24056 (7) Å	b = 16.2359 (2) Å
	c = 6.13775 (3) Å	c = 6.13160 (10) Å
	*α* = 90°	*α* = 90°
	*β* = 90°	*β* = 90°
	*γ* = 90°	*γ* = 90°
Cell Volume	V = 2,705.74 (2) Å^3^	V = 2,704.02 (7) Å^3^
Cell Formula Units	Z = 4	Z = 4
Crystal Density	D_c_ = 1.374 Mg/m^3^	*D*_*c*_ = 1.375 Mg/m^3^
Crystal F (000)	1,184.0	1,184.0
Absorption Coefficient mu	μ(Cu Kα) = 1.613 mm^−1^	μ(Cu Kα) = 1.614 mm^−1^
Limiting Indices	-32 ≤ *h* ≤ 32	-32 ≤ *h* ≤ 30
	-19 ≤ k ≤ 19	-19 ≤ k ≤ 16
	-7≤ l ≤ 6	-7≤ l ≤ 7
Cell Measurement Temperature	T = 99.99 (11) K	T = 99.99 (11) K
2θ range for data collection	6.342–133.2°	6.342–133.2°
Goodness-of-fit on F^2	1.046	1.041
Final R indices [I > 2sigma(I)]	R_1_ = 0.0318, wR2 = 0.0828	R_1_ = 0.0324, wR_2_ = 0.0824
R indices (all data)	R_1_ = 0.0337, wR2 = 0.0854	R_1_ = 0.0343, wR_2_ = 0.0854
Largest diff. peak and hole	0.26 and -0.32 e.Å^−3^	0.32 and -0.26 e.Å^−3^
Reflections collected	48,459	36,655
Independent Reflections	4803 [R_(int)_ = 0.0672]	4793 [R_(int)_ = 0.0525]
Flack parameter	-0.012 (6)	-0.010 (7)

### VMAT2 Binding Assay

The affinities of 13e, (+)-13e, and (−)-13e for VMAT2 were measured as the inhibition of [^3^H]HTBZ binding to rat brain VMAT2. As presented in Table 2, (+)-13e had greater affinity (*K*_i_ = 1.48 nM) for VMAT2 than (−)-13e (*K*_i_ = 270 nM). In addition, the affinity of (+)-13e for VMAT2 was nearly 3-fold stronger than that of HTBZ (*K*_i_ = 4.22 nM).

**TABLE 2:** *In vitro* vesicular monoamine transporter 2 (VMAT2) binding affinity in the rat brain and inhibition of [^3^H]DA uptake in rat striatal synaptosomes.

Compounds	Binding (nM)		Uptake (nM)	
	IC_50_	K_i_	IC_50_	IC_90_
13e	5.45	3.18	7.89	36.7
(+)-13e	2.54	1.48	6.11	34.1
(−)-13e	470	270	129	1,598
HTBZ	7.23	4.22	30.6	277
TBZ	23.0	13.5	7.17	96.9

### Inhibition of [^3^H]DA Uptake

The functionality of both isomers was determined using an *in vitro* [^3^H]DA uptake assay in rat striatal synaptosomes (Table 2). The results demonstrated that (+)-13e exhibited strong ability [^3^H]DA uptake by VMAT2, whereas the inhibitory effects of (−)-13e were weak (IC_50_ = 129 nM). The IC_50_ values of (+)-13e and HTBZ regarding the dopamine transport function of rat VMAT2 were 6.11 and 30.61 nM, respectively, indicating that the inhibitory effect of (+)-13e was approximately 5-fold stronger than that of HTBZ.

### Dose-dependent Effects on the Locomotor Activity in Rats

**FIGURE 3:**
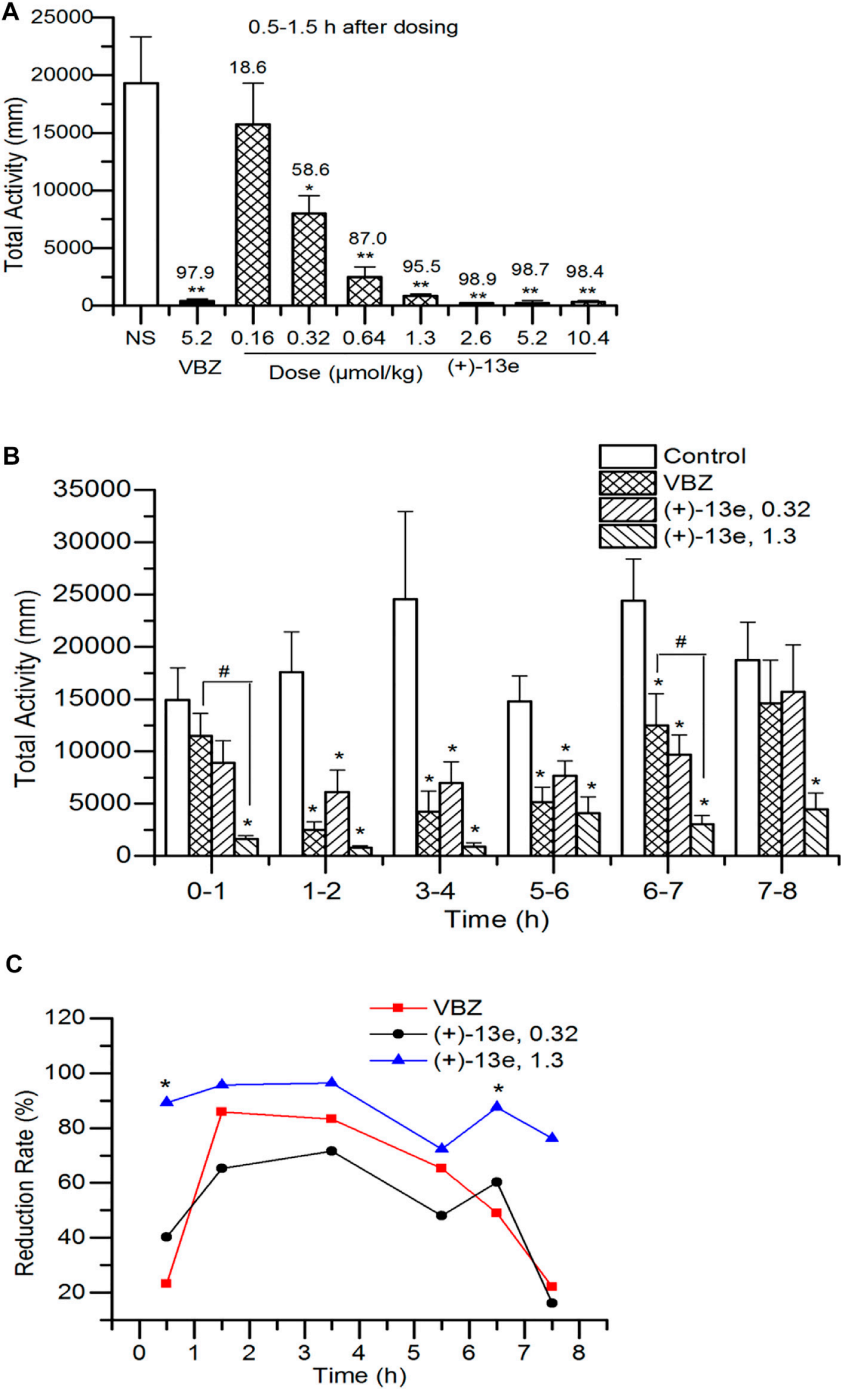
Locomotor activity in Sprague–Dawley rats after a single intragastric dose of (+)-9-trifluoroethoxy-α-dihydrotetrabenazine [(+)-13e] **(A)** Dose-dependent effects were assessed using the distance of locomotion at 0.5–1.5 h after administration, and the upper value of the histogram was the reduction rate of locomotion (%) **(B)** Time-dependent effects were evaluated using the distance of locomotion at different periods after administration of (+)-13e at 0.32 and 1.3 μmol/kg, taking valbenazine (VBZ) as control at oral dosing of 1.3 μmol/kg **(C)** The time–effect relationship of the reduction rate of locomotion. The results are expressed as the mean ± SEM, n = 12/group. ^*^ *p* < 0.05, ^**^ *p* < 0.01, compared with the NS group. ^#^ *p* < 0.05, compared with VBZ group.

The total distance traveled by the rats was recorded for 1 h starting 0.5 h after the administration of different doses of (+)-13e. The results (Figure 3A) indicated that in the dose range of 0.16–10.4 μmol/kg, (+)-13e inhibited the locomotor activity of rats in a dose-dependent manner (RR = 18.6–98.9%). Except for the 0.16 μmol/kg dose group, significant inhibition of rat locomotor activity was observed in all test groups compared with that in the NS group (*p* < 0.05). The starting effective dose was 0.32 μmol/kg (RR = 58.6%), and at a dose of 1.3 μmol/kg, (+)-13e exhibited excellent inhibitory effects (RR = 95.5%). As a positive control, VBZ (5.2 μmol/kg) also displayed similar efficacy (RR = 97.9%). At the three higher doses of (+)-13e (2.6, 5.2 and 10.4 μmol/kg), the inhibitory effects on the locomotor activity of rats reached a plateau at RR > 98.4%.

### Time-dependent Effects on Locomotor Activity in Rats

The time–effect relationship of (+)-13e was investigated at doses of 0.32 and 1.3 μmol/kg based on the aforementioned results, and the distances traveled were recorded for rats in different periods (0–1, 1–2, 3–4, 5–6, 6–7, and 7–8 h) after administration. The results (Figure 3B) indicated that excluding the first and last periods, the distances traveled were significantly reduced by (+)-13e at 0.32 μmol/kg and VBZ at 1.3 μmol/kg at other period (*p* < 0.05 vs the NS control) (+)-13e at 1.3 μmol/kg inhibited locomotion in rats in all periods (*p* < 0.05 vs the NS control), indicating that (+)-13e has faster and longer-lasting effects than VBZ at an equivalent dose, and the RR of (+)-13e (72.3%–96.4%) was larger than that of VBZ (22.8–85.9%) in tested period, and the differences were significant at 0–1 h and 7–8 h (*p* < 0.05, Figure 3C).

### Bidirectional Permeability in Caco-2 Cell Monolayers

**FIGURE 4:**
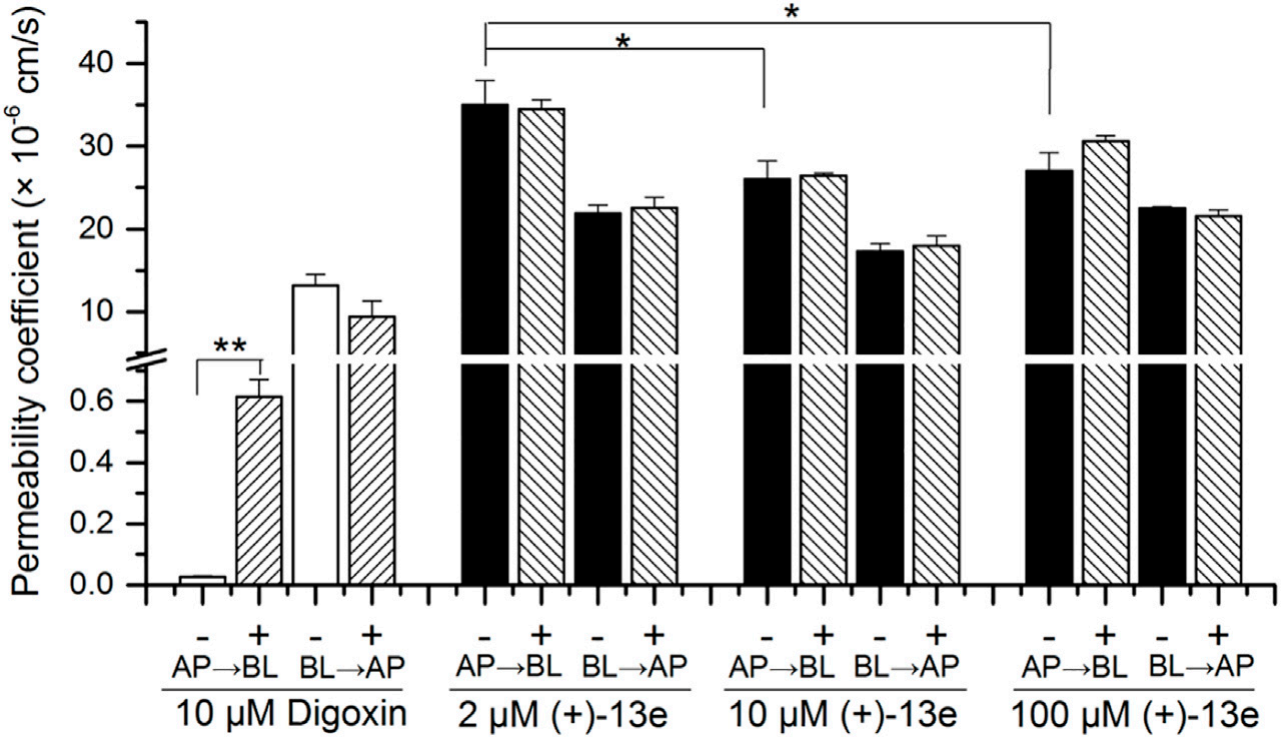
Bidirectional permeability of (+)-13e in Caco-2 cell monolayers. HBSS containing (+)-13e (2, 10 and 100 μM) or digoxin (10 μM) was loaded into either the apical (AP) or basolateral (BL) chambers with (+) or without (-) verapamil (30 μM, P-gp inhibitor) for 2 h incubation at 37 °C. Data are presented as the mean ± SD of three independent experiments. ^*^ *p* < 0.05, compared with the 10 and 100 µM concentration groups. ^**^ *p* < 0.01, compared with the presence of verapamil.

The result (Figure 4) demonstrated that in the presence or absence of verapamil, an inhibitor of P-glycoprotein (P-gp), the mean P_app_ (AP→BL) values of (+)-13e at concentrations of 2.0, 10.0, and 100 µM ranged 26.0 × 10^−6^–35.0 × 10^–6^ cm/s, and the value at 2 µM was slightly higher (1.3 times) than that at 10 and 100 µM (*p* < 0.05). The P_app_ values of (+)-13e in both AP→BL and BL→AP directions were not affected by verapamil (*p* > 0.05) at the tested concentrations, and the ER ranged from 0.626 to 0.833. Meanwhile, the P_app_ (AP→BL) values of digoxin (a substrate of P-gp) was increased by 22 times (*p* < 0.01) when verapamil was present. Therefore, (+)-13e demonstrated high permeability across the Caco-2 cell monolayer, and efflux was not observed, indicating that (+)-13e is unlikely to be a substrate of P-gp or breast cancer resistance protein (BCRP).

### Metabolite Identification

#### Metabolite Identification in Liver Microsomes

**FIGURE 5:**
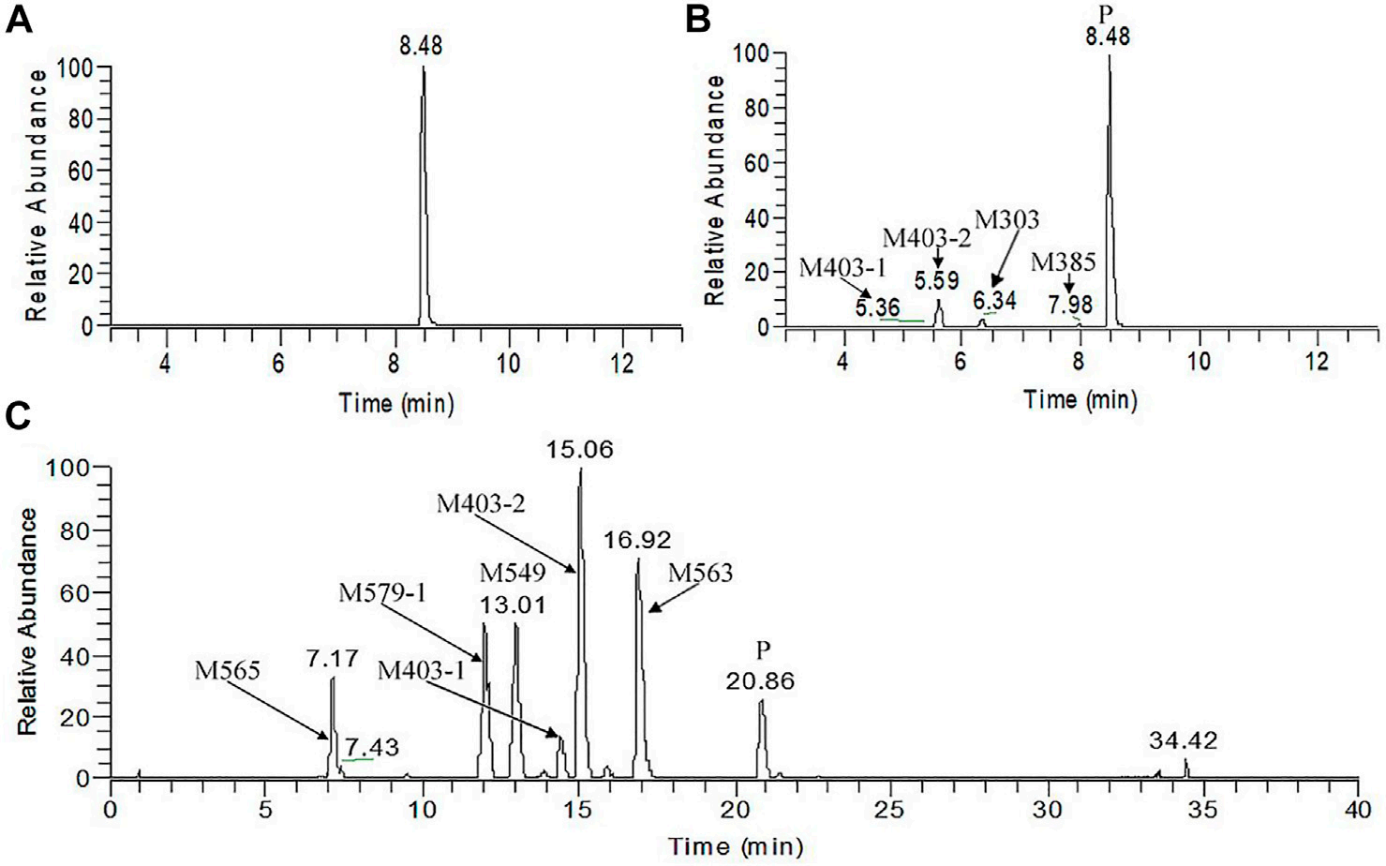
The extracted ion chromatograms of human liver microsomes incubated without **(A)** and with NADPH **(B)** and dog plasma samples obtained after a single oral dose of (+)-9-trifluoroethoxy-α-dihydrotetrabenazine at 3 μmol/kg **(C)**.

**FIGURE 6:**
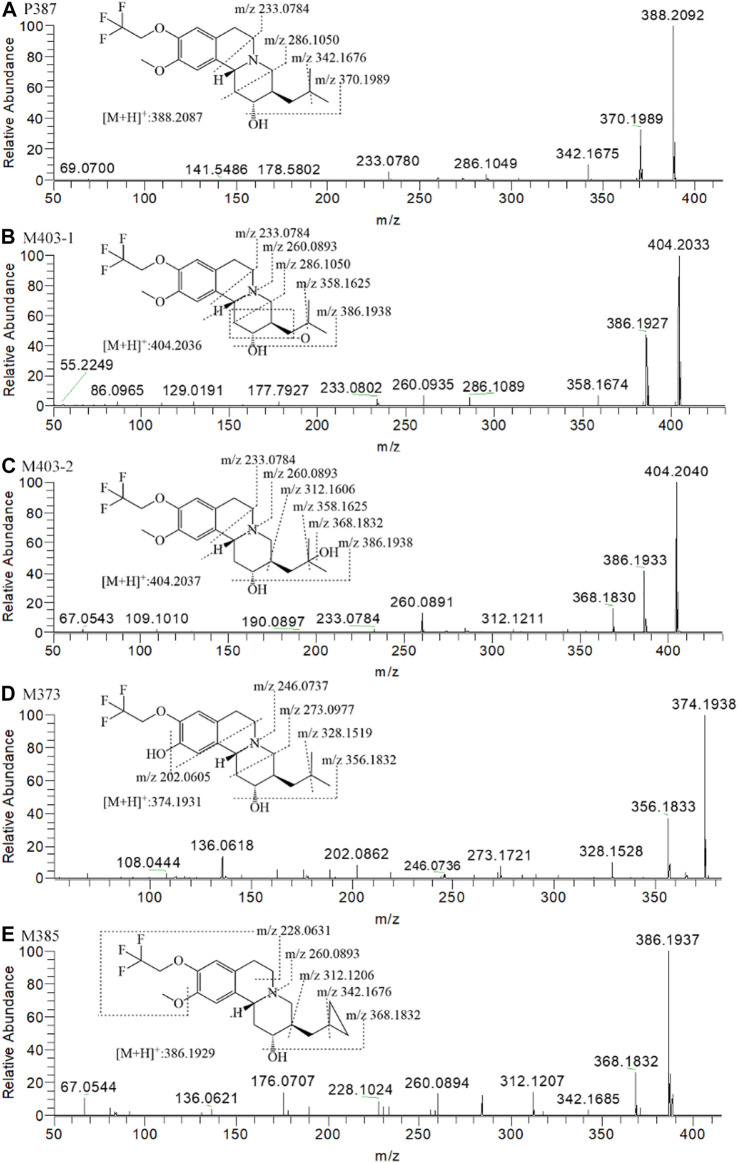
The MS^2^ spectra and proposed fragmentation pathways of the metabolites of (+)-9-trifluoroethoxy-α-dihydrotetrabenazine [(+)-13e] after incubation in human liver microsomes.

Figure 5A, B presents the typical extracted ion chromatograms and retention times of (+)-13e and its detected metabolites in HLMs (+)-13e was eluted at 8.48 min with an [M + H]^+^ ion at *m/z* 388.2087 (−1.8 ppm) in the positive ion mode (Figure 6A). As presented in Figure 5B, four metabolites were identified through our analytical strategy, including two mono-oxidation metabolites (M403-1, M403-2), one demethylation metabolite (M373), and one dehydrogenation metabolite (M385). Table 3 lists the mass spectra and relative abundance of the identified (+)-13e metabolites, indicating that the parent drug accounted for the main proportion of the content, with a relative abundance of 85.44–88.72% in liver microsomes from humans, dogs, and rats. In addition, M403-2 was the main metabolic product, with a relative abundance of 8.78–12.11%. The relative abundances of M403-1, M373, and M385 were all less than 3.2%. The results demonstrated robust metabolism stability at the nine position, and O-detrifluoroethylation did not occur in liver microsomes from humans, rats, and dogs. Hence, we speculate that (+)-13e has a long active half-life.

**TABLE 3:** Summary of the metabolites of (+)-9-trifluoroethoxy-α-dihydrotetrabenazine [(+)-13e] detected in human, dog and rat liver microsomes and in dog plasma using the UPLC-Q Exactive™ Orbitrap HRMS.

No	TR min	Theonetical Mass m/z	Experimental Mass m/z	Error ppm	Fomula [M + H]^+^	Fragment ions m/z	Identification/Reaction	Relative abundance, UV area %		
								Human	Dog	Rat
Liver microsomes										
M403-1	5.36	404.2043	404.2036	-1.7	C_20_H_29_F_3_NO_4_	386, 358, 286, 260, 233	Mono-oxidation	0.72^*^	1.27	0.48^*^
M403-2	5.59	404.2043	404.2037	-1.5	C_20_H_28_F_3_NO_4_	386, 368, 312, 260, 233	Mono-oxidation	12.11	10.01	8.78
M373	6.34	374.1938	374.1931	-1.9	C_19_H_26_F_3_NO_3_	356, 328, 273, 246	Demethylation	0.69^*^	0.46^*^	2.6
M385	7.98	386.1938	386.1929	-2.3	C_20_H_26_F_3_NO_3_	368, 342, 312, 260	Dehydrogenation	1.02	1.15^*^	3.18
P	8.48	388.2094	388.2087	-1.8	C_20_H_29_F_3_NO_3_	370, 342, 286, 233	NA	85.31	87.01	84.91
Dog plasma							Identification/Reaction			UV area %
M565	7.17	566.2208	566.2203	-0.9	C_25_H_34_O_10_NF_3_	548, 390, 372, 354, 298, 246	Mono-oxidation,demethylation andglucuronidation			10.2
M579-1	12.04	580.2364	580.2355	-1.6	C_26_H_36_O_10_NF_3_	562, 404, 386, 368, 312, 260	Mono-oxidation andglucuronidation			3.43
M579-2	12.88	580.2364	580.2357	-1.2	C2_6_H_36_O_10_NF_3_	404, 386, 358, 260	Mono-oxidation andglucuronidation			+
M549	13.01	550.2258	550.2251	-1.3	C_25_H_34_O_9_NF_3_	532, 374, 356, 328, 273, 219	Demethylation andglucuronidation			7.47
M403-1	14.46	404.2043	404.2038	-1.2	C_20_H_28_O_4_NF_3_	386, 358, 286, 260, 233	Mono-oxidation			1.47
M403-2	15.06	404.2043	404.2039	-1.0	C_20_H_28_O_4_NF_3_	386, 368, 312, 260, 233	Mono-oxidation			9.69
M563	16.92	564.2415	564.2409	-1.1	C_26_H_36_O_9_NF_3_	388, 370, 342, 286, 233	Glucuronidation			9.92
P	20.86	388.2094	388.2089	-1.3	C_20_H_28_O_3_NF_3_	370, 342, 286, 260, 233	NA			57.3

*, It is the relative abundance detected by mass spectrometry, and there is no the peak in UV, detection, and the relative abundances of other peaks detected by the two detectors are similar.

Theoretical Mass (*m/z*) = Exact Mass +1.0073 (H^+^); Exact Mass: Monoisotopic Mass; Error (ppm) = [(Measured Mass - Theoretical Mass) ÷Theoretical Mass]×10^6^; NA: not applicable.

According to the fragment ion mass analysis of (+)-13e and its metabolites, as presented in Figures 6A–E and Table 3, the main metabolite M403-2 was oxidized at the angular methyl of the isobutyl at the three position because there no characteristic fragment ion at *m/z* 358 compared to M403-1, which featured the loss of two angular methyl groups to form a fragment ion at *m/z* 358. O-demethylation occurred at the 10 position to form M373, and M385 was produced via dehydrogenation of the isobutyl to form a cyclopropylmethyl based on the presence of the characteristic fragment ion at *m/z* 342, which matched the fragment ion produced when the two angular methyl groups were removed from (+)-13e. In addition, the mass loss was 26 compared to the presence of the adjacent heavier fragment ion.

#### Metabolite Identification in Dog Plasma

**FIGURE 7:**
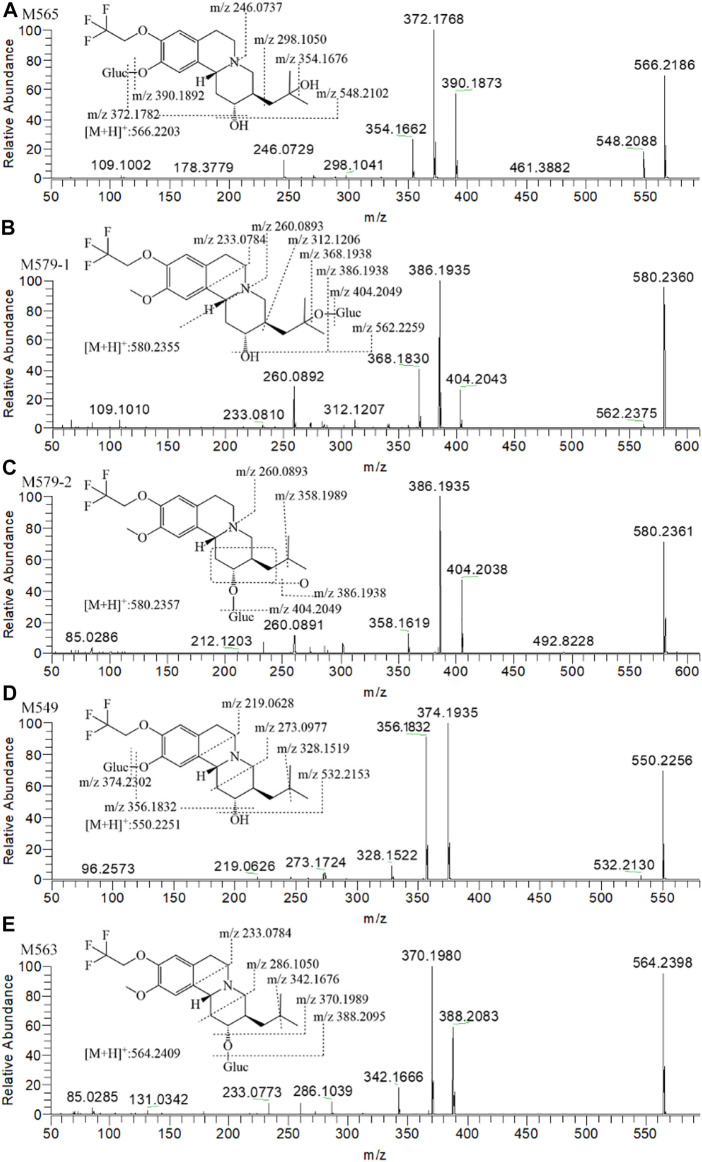
The MS^2^ spectra and proposed fragmentation pathways of (+)-9-trifluoroethoxy-α-dihydrotetrabenazine [(+)-13e] metabolites in dog plasma after a single oral dose at 3 μmol/kg.

Figure 5C presents the extracted ion chromatograms and retention times of (+)-13e and its detected metabolites in dog plasma after oral administration. We found that (+)-13e was metabolized to two mono-oxidized products (M403-1, M403-2); one mono-oxidized, demethylated, and glucuronidated product (M565); two mono-oxidized and glucuronidated products (M579-1, M579-2); one demethylated and glucuronidated product (M549); and one glucuronide conjugate (M563) in dog plasma. The MS^2^ spectra of M403–1 and M403-2 in dog plasma were identical to those in HLMs (Figures 6B,C). Figures 7A–E presents the MS^2^ spectra of other metabolites and the proposed fragmentation pathways. Table 3 also lists the mass spectrum information of the (+)-13e metabolites identified in dog plasma and their relative abundances calculated from the UV peak areas, indicating that (+)-13e accounted for most of the content (relative abundance of 57.3%). Meanwhile, M565, M563, M403-2, and M549 were the main metabolites, with relative abundances of 10.2, 9.92, 9.69, and 7.47%, respectively. The relative abundances of M579-1 and M403-1 were 3.43 and 1.47%, respectively.

Combined with the metabolic pathways of (+)-13e identified in liver microsomes and identified metabolites in dog plasma, we speculated that (+)-13e undergoes three routes of metabolism *in vivo*, namely mono-oxidation, demethylation, and glucuronide conjugation, with some metabolites undergoing multiple processes (Figure 1C). In these metabolic pathways, the metabolism of O-detrifluoroethylation at the nine position did not occur, demonstrating robust stability at this position.

### Metabolic Phenotype by P450 Enzymes

According to the metabolite identification results, M403, M373, and M385 were selected as the targets for metabolic phenotyping using chemical inhibitors in HLMs and rhCYP isozymes. As presented in Table 4, the inhibition rates of the seven probe substrates in HLMs and the clearance data of the probe substrates with rhCYP isozymes were all normal. The experiment in HLMs using chemical inhibitors demonstrated that the mono-oxidation, demethylation, and dehydrogenation of (+)-13e were mainly mediated by CYP3A4, as ketoconazole (a CYP3A4 inhibitor) inhibited these processes by 91.5, 80.8, and 91.3%, respectively. The secondary metabolic enzyme was CYP2C8. In the presence of the CYP2C8 inhibitor montelukast, mono-oxidation, demethylation, and dehydrogenation were inhibited by 56.2, 22.3, and 50.9%, respectively. The experiment with rhCYP isozymes indicated that CYP3A4 is the primary isozyme responsible for the mono-oxidation, demethylation, and dehydrogenation of (+)-13e, and the relative contributions of CYP3A4 ranged from 93.2 to 96.7%. Meanwhile, other isozymes had little to no contribution.

**TABLE 4:** Relative contribution of individual cytochrome P450 isoforms to generation of the metabolites M403 (mono-oxidized product), M373 (demethylated product), and M385 (dehydrogenated product) from (+)-9-trifluoroethoxy-α-dihydrotetrabenazine [(+)-13e] in human liver microsomes and recombinantly expressed cytochrome P450 (rhCYP) isozymes.

Isoenzyme	Specific substrates				(+)-13e					
	Kinds	Final conc. (µM) HLM/rhCYP	%Inhibation in HLM	*CL*_int_ in rhCYP (µL/min/pmol)	% Inhibition in HLMs			% Relative Contribution by rhCYP		
					M403	M373	M385	M403	M373	M385
CYP1A2	Phenacetin	25/5	87.7	51.8	13.4	-13.3	14.5	3.3	0	3.3
CYP2B6	Bupropion	100/20	90.5	31.2	-13.1	-0.97	-14.0	0	0	0
CYP2C8	Amodiaquine	2/0.4	90.8	17.7	56.2	22.3	50.9	0	1.1	0
CYP2C9	Diclofenac	5/1	89.9	6.15	19.6	18.0	1.3	0	3.3	0
CYP2C19	(S) -Mephenytoin	40/8	79.0	2.99	-6.4	-1.3	-4.8	0.4	0.7	0
CYP2D6	Dextromethorphan	5/1	90.7	13.1	-7.2	7.7	-18.8	0.3	1.7	0
CYP3A4	Midazolam	2/0.4	84.7	9.13	91.5	80.8	91.3	96.0	93.2	96.7

### Pharmacokinetics in Dogs

The PK parameters of (+)-13e in dogs after a single oral or intravenous dose are listed in Table 5 (+)-13e was absorbed rapidly after administration, and the mean peak concentration (C_max_) was 507 nM with a peak time of 0.75 h, and its plasma elimination half-life was 6.8 h. The absolute oral bioavailability of (+)-13e was 75.9%. C_max_ of (+)-13e was 2.9-fold smaller than that of VBZ and 7.7-fold larger than that of HTBZ after oral administration at an equivalent dose. Moreover, although the oral bioavailability of VBZ was 74.1%, the percent molar ratios of HTBZ in dogs after oral and intravenous administration were 6.3 and 4.1%, respectively. Therefore, (+)-13e displayed excellent PK properties.

**TABLE 5:** The pharmacokinetic parameters of (+)-9-trifluoroethoxy-α-dihydrotetrabenazine [(+)-13e], valbenazine (VBZ), and its active metabolite (+)-α-dihydrotetrabenazine (HTBZ) in dog plasma after a single oral dose at 3 μmol/kg (n = 4) and single intravenous dose of 0.6 μmol/kg (n = 3).

Parameters	Oral			Intravenous		
	(+)-13e	VBZ	HTBZ	(+)-13e	VBZ	HTBZ
T_max_ (h)	0.75 ± 0.43	1.4 ± 0.8	3.3 ± 0.6	0.083 ± 0	0.083 ± 0	0.83 ± 0.3
C_0_, C_max_ (nM)	507 ± 98	1,465 ± 461	66.1 ± 58.7	382 ± 148	747 ± 118	14.1 ± 5.1
AUC_last_ (h·nM)	1,610 ± 386	6,224 ± 1,074	524 ± 475	424 ± 15.8	1,679 ± 365	61.1 ± 21.4
AUC_inf_ (h·nM)	1,680 ± 394	6,385 ± 1,101	607 ± 532	441 ± 20.7	1722 ± 384	76.8 ± 24.2
V, V/F (L/kg)	18.8 ± 9.2	3.5 ± 0.8	-	5.4 ± 1.1	2.8 ± 0.5	
Cl, Cl/F (L/kg/h)	1.9 ± 0.4	0.48 ± 0.08	-	1.4 ± 0.1	0.36 ± 0.07	
T_1/2_ (h)	6.8 ± 2.0	5.1 ± 0.8	5.3 ± 2.6	2.8 ± 0.7	5.5 ± 1.3	2.8 ± 0.3

T_max_: time to C_max_; C_0_: initial concentration after intravenous administration; C_max_: peak concentration; AUC_last_: area under the concentration-time curve from time zero to the last measurement; AUC_inf_: area under the concentration-time curve from time zero to infinity; V: apparent distribution volume; Cl: clearance; F: bioavailability; T_1/2_: terminal elimination half-life.

## Discussion

Because VMAT2 plays a critical role in dopamine transport, inhibition of VMAT2 activity could be a promising strategy for the treatment of movement disorders (Lohr et al., 2017). Specifically, VMAT2 inhibitors may be useful in offsetting the movement-related side effects of antipsychotics and other dopamine receptor-blocking agents (Jankovic, 2016). Although TBZ is the first VMAT2 inhibitor, initially used as an agent to ameliorate symptoms of TD, and DTBZ retains the VMAT2 affinities of the non-deuterated isomers, TBZ/DTBZ is a racemate of two ketone enantiomers, and its metabolism is complex, illustrated in Figure 1A. After carbonyl reductase reduces, four stereoisomers of dihydrotetrabenazine were formed, (-)-α-HTBZ and (+)-β-HTBZ were the most abundant isomers (Skor et al., 2017; Stahl, 2018). Although the (+)-α-HTBZ is the most potent and selective inhibitor of VMAT2 among the metabolite isomers of TBZ (Yao et al., 2011; Grigoriadis et al., 2017), its concentration in the blood is quite low. Instead, (+)-β-HTBZ, a less potential VMAT2 inhibitor, has much higher concentrations. Both (−)-α-HTBZ and (−)-β-HTBZ has much low potency for VMAT2 inhibition and may contribute to off-target effects of TBZ and DTBZ.

In our study, (+)-13e, a high-potential VMAT2 inhibitor (*K*_i_ = 1.48 nM), was acquired via chiral separation (−)-13e has weak inhibition on VMAT2 activity with similar potency as (−)-β-HTBZ (Grigoriadis et al., 2017) (+)-13e displayed greater affinity for VMAT2 and stronger inhibitory effects on [^3^H]DA uptake by VMAT2 than the positive control HTBZ. In molecular modeling experiments, the trifluoroethyl substituent was demonstrated to increase the efficiency of binding to the target protein (Massa et al., 2001). In a structure–activity relationship study of dihydrotetrabenazine derivatives, it was found that the derivatives had better inhibitory effects on VMAT2 when the chain length of the substituent at position nine was greater than that at the 10 position (Yang et al., 2021).

It has been reported an inactive metabolite of HTBZ is formed by demethylation at position 9, which is the more prevalent O-demethyl metabolism site, and CYP2D6 plays a main role in this process (Harriott et al., 2018; Schneider et al., 2020). For VBZ, TBZ, and DTBZ, CYP2D6 plays a major role in the formation of desmethylated metabolites. Poor metabolizers will have 2–3-fold higher active metabolite concentrations than those with normal CYP2D6 metabolism, which may influence the risk for TD (Jankovic, 2016; Scorr and Factor, 2018). A number of examples from the literature (Böhm et al., 2004; Singh, 2006) illustrate that the replacement of C-H by C-F enhances metabolic stability. In our previous study (Yang et al., 2021), 13e was more stable than HTBZ in liver microsomes from different species, with a 2.4–4.8-fold decrease of clearance. We expected the introduction of a trifluoroethyl group at the nine position to block the metabolically labile spots, thereby prolonging the active half-life and avoiding the polymorphic effects of CYP2D6. Indeed, robust metabolism stability at the nine position demonstrated by *in vitro* and *in vivo* metabolite identification experiments, and O-detrifluoroethylation did not occur in liver microsomes and dog plasma after oral adiministration. Hence, we speculate that (+)-13e has a long active half-life, which should prolong efficacy *in vivo*. In addition, CYP2D6 polymorphism does not affect (+)-13e, so a “tailor-made” drug for each individual is unnecessary. It was further confirmed in the metabolic enzyme phenotype experiment in this study that CYP3A4 is the main isozyme responsible for the metabolism of (+)-13e, and CYP2C8 is a secondary isozyme.

The introduction of a fluorine atom into a molecule can be used to modulate physicochemical properties such as lipophilicity and basicity (Böhm et al., 2004). It was reported that mono-fluorination or trifluoromethylation of saturated alkyl groups usually decreases lipophilicity because of the strong electron-withdrawing capabilities of the fluorine (Purser et al., 2008). Hence, we evaluated the permeability of (+)-13e using Caco-2 cells, a widely used *in vitro* model for small intestinal absorption with positive P-gp and BCRP expression. The result indicated that (+)-13e has high permeability and that it is not a substrate of P-gp and BCRP, which should permit good absorption *in vivo*. We evaluated the *in vivo* PK of (+)-13e and identified its metabolites after a single oral dose in dogs, as their metabolic pathways are mediated by intestinal microbiota and P450s closely resemble those in humans (Coelho et al., 2018). The absolute bioavailability and longer elimination half-life of (+)-13e indicated that it was well absorbed after oral administration and had good metabolism stability. Therefore, (+)-13e displayed excellent PK properties.

The aforementioned PK characteristics are undoubtedly helpful for obtaining good PD results *in vivo*. VMAT2 inhibitors can inhibit the release of dopamine and reduce rat locomotor activity, which is used as a substitute index to evaluate the efficacy of VMAT2 inhibitors (Grigoriadis et al., 2017; Guzen et al., 2019; Li et al., 2019). The multi-target activity screen of SpectrumScreen^®^ Panel (Eurofins screen) demonstrated that (+)-13e had no effects on dopamine receptors, serotonin receptors, adrenergic receptors, dopamine transporter, norepinephrine transporter, five serotonin transporter, and so on. Therefore, any activity in the rat autonomous activity model is likely due to inhibition of VMAT2 resulted in monoamine depletion as opposed to, for example, dopaminergic D_2_ receptor antagonism. The PD effect of (+)-13e was approximately 4-fold stronger than that of VBZ *in vivo*, and time–effect experiment indicated that (+)-13e provides faster and longer-lasting effects than VBZ at an equivalent dose. In our previous study, the concentration of 13e in the brain was 2-fold higher than that of HTBZ and the concentrations of 13e in the heart was approximately half that of VBZ after oral administration at an equimolar dose in SD rats, supporting our results. In addition, VBZ and 13e blocked the hERG current with IC_50_ values of 1.7 and 6.51 nM, respectively (Yang et al., 2021). Therefore, we anticipate that (+)-13e has no potential to cause Q-T prolongation based on its decreased dose and peak concentration.

## Conclusion

In this study, we acquired the 3R configuration isomer (+)-13e and its enantiomer via chiral resolution of 13e and demonstrated that (+)-13e has superior VMAT2-binding affinity and inhibitory effects on [^3^H]DA uptake than HTBZ (+)-13e decreased the locomotor activity of rats in a dose-dependent manner, and a significant effect was first observed at a dose of 0.32 μmol/kg. In addition, significant inhibition was sustained for at least 8 h at a dose of 1.3 μmol/kg as a result of its excellent PK properties.

(+)-13e mainly undergoes mono-oxidation, demethylation, and glucuronide conjugation *in vivo*. CYP3A4 is the primary isozyme responsible for mono-oxidation and demethylation, and CYP2C8 is a secondary isozyme. A trifluoroethyl substitution prevented O-dealkylation at the nine position, which occurs in HTBZ (production of inactive metabolite), and CYD2D6 polymorphism had no effect on the PK of (+)-13e (+)-13e had high permeability, and it was not a substrate of P-gp and BCRP. These improved PD and PK properties may enable the administration of (+)-13e at lower doses, resulting in lower risks of side effects (e.g., Q-T prolongation). Thus, (+)-13e deserves further development as a new treatment for TD.

## Data Availability

The datasets presented in this study can be found in online repositories. The names of the repository/repositories and accession number(s) can be found below: https://www.ccdc.cam.ac.uk/structures/, accession ID: 2110605 and 2110606
